# Multi-Weather DomainShifter: A Comprehensive Multi-Weather Transfer LLM Agent for Handling Domain Shift in Aerial Image Processing

**DOI:** 10.3390/jimaging11110395

**Published:** 2025-11-06

**Authors:** Yubo Wang, Ruijia Wen, Hiroyuki Ishii, Jun Ohya

**Affiliations:** Department of Modern Mechanical Engineering, Waseda University, Tokyo 169-8555, Japan

**Keywords:** aerial image processing, domain shift, semantic segmentation, style transfer, image generation, synthetic data, large language model agent

## Abstract

Recent deep learning-based remote sensing analysis models often struggle with performance degradation due to domain shifts caused by illumination variations (clear to overcast), changing atmospheric conditions (clear to foggy, dusty), and physical scene changes (clear to snowy). Addressing domain shift in aerial image segmentation is challenging due to limited training data availability, including costly data collection and annotation. We propose Multi-Weather DomainShifter, a comprehensive multi-weather domain transfer system that augments single-domain images into various weather conditions without additional laborious annotation, coordinated by a large language model (LLM) agent. Specifically, we utilize Unreal Engine to construct a synthetic dataset featuring images captured under diverse conditions such as overcast, foggy, and dusty settings. We then propose a latent space style transfer model that generates alternate domain versions based on real aerial datasets. Additionally, we present a multi-modal snowy scene diffusion model with LLM-assisted scene descriptors to add snowy elements into scenes. Multi-weather DomainShifter integrates these two approaches into a tool library and leverages the agent for tool selection and execution. Extensive experiments on the ISPRS Vaihingen and Potsdam dataset demonstrate that domain shift caused by weather change in aerial image-leads to significant performance drops, then verify our proposal’s capacity to adapt models to perform well in shifted domains while maintaining their effectiveness in the original domain.

## 1. Introduction

Aerial Image Segmentation (AIS) is an essential task for various city monitoring purposes, such as environmental surveillance, target localization, and disaster response [1,2,3]. With semantic segmentation models trained on large-scale annotated data, humans can easily extract abundant geo-spatial information from aerial images captured by drones or satellites [4,5,6].

### 1.1. The Challenge of Weather Change-Caused Domain Shift in Aerial Imagery

**Figure 1:**
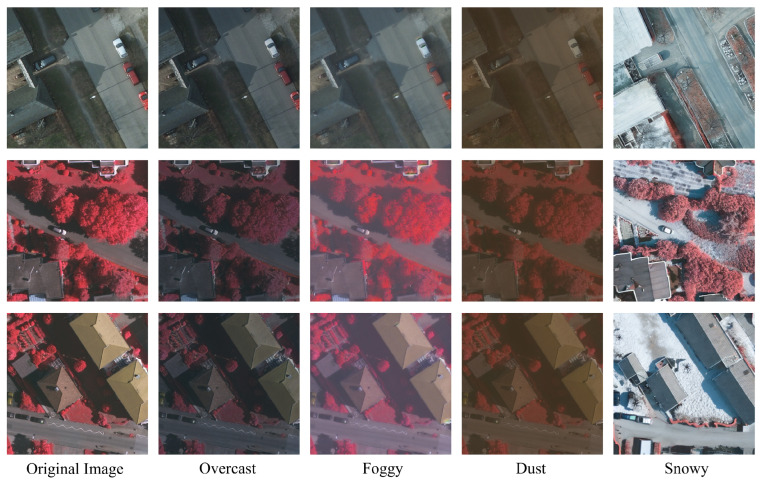
Examples of domain shift in aerial images across multiple weather conditions. From left to right, the columns show original, overcast, foggy, dusty, and snowy conditions. Each row presents different aerial samples from ISPRS datasets [10,11]. (High-resolution figure, zoom in for a better view).

While the performance of semantic segmentation algorithms has surged on common benchmarks, progress in handling the domain shift of unseen environmental conditions is still stagnant [7,8,9]. We demonstrate that the aerial segmentation performance of algorithms is prone to significant degradation due to *Domain Shift*, i.e., the transfer from one domain to another. In Figure 1, we illustrate this phenomenon by comparing the original data in the ISPRS datasets [10,11] with our generated domain-shifted versions, including illumination variations (clear to overcast), changing atmospheric conditions (clear to foggy, dusty), and physical scene changes (clear to snowy). Notably, the scene content and target information remain consistent across all weather variations, while atmospheric conditions and illumination characteristics change significantly in the first three weather domains. The last snowy condition presents additional challenges with physical scene changes including leaf drop, snow-covered roofs, and ground while preserving the information of targets of interest. This figure demonstrates the challenge of cross-domain generalization in aerial image analysis.

**Figure 2:**
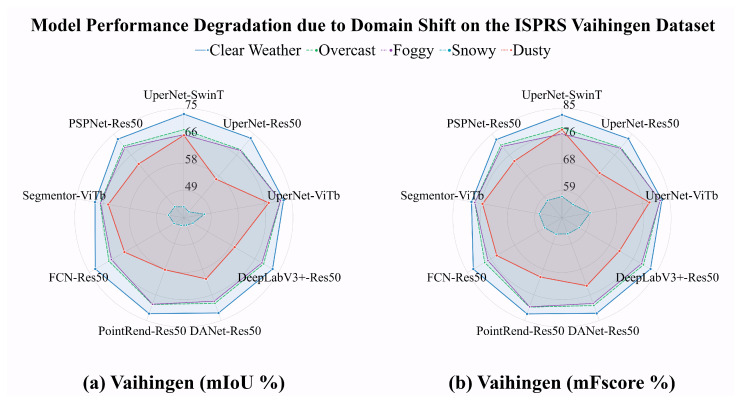
Performance degradation due to domain shift on the ISPRS Vaihingen datasets. The figure illustrates model performance on the Vaihingen datasets, reporting both mIoU (**a**) and mFscore (**b**) metrics. We pre-trained nine prevalent segmentation models with various backbones on the original training set: UperNet with three different backbones (Swin Transformer [12], ResNet-50 [13], and ViT-Base [14]), DeepLabV3Plus-ResNet-50, DANet-ResNet-50, PointRend-ResNet-50, FCN-ResNet-50, Segmentor-ViT-Base, and PSPNet-ResNet-50 [15,16,17,18,19,20,21]. We then tested them on both the original validation set (clear weather, solid blue lines) and our generated domain-shifted validation sets under various weather conditions (dashed lines in different colors). The results demonstrate significant performance deterioration caused by domain shift compared to the original performance under clear weather. (High-resolution figure, zoom in for a better view).

The radar chart in Figure 2, in which our evaluations of nine state-of-the-art segmentation models on the ISPRS dataset [10,11] and its domain-shifted version are shown, further illustrates that even within the same scene and objects, slightly altering the atmospheric conditions and varying lighting levels pose challenges for aerial image segmentation algorithms. As detailed in the caption of Figure 2, the results show that after transferring the data from its original, intact domain to shifted domains, there is an average mIoU deterioration of {**−3.35%**, **−3.92%**, **−10.55%**, **−28.59%**} and mFscore deterioration of {**−2.61%**, **−3.24%**, **−8.66%**, **−25.76%**} under overcast, foggy, dusty, and snowy conditions on the Vaihingen dataset (512×512 resolution). Notably, we generated snowy image sets with five different random seeds, and the numeric results represent the average across these five sets. Compared to the original intact data, the illumination in the shifted overcast images is reduced; foggy and dusty weather additionally changes the atmospheric information, and the snowy scene add physical changes to the target of interest in the original scene, representing typical domain shift. However, the image content, layout, and geo-spatial information between the original and shifted data remain unchanged.

Closing the gap between model performance in the original domain and the shifted domain is a valuable problem to address. An intuitive solution is to incorporate multi-domain data into the model training process. The performance of aerial image segmentation models significantly relies on the availability of training data. Although data from adverse domains is essential to improve the robustness of aerial image segmentation models, such data—including aerial images captured under low illumination and harsh weather conditions—is lacking in the current aerial image benchmarks [10,11,22]. On the other hand, even if sufficient data is obtained, annotation remains a time-consuming and labor-intensive task. This paper breaks the limitation of low-domain diversity while eliminating the need for additional annotations on shifted domain data.

### 1.2. Recent Developments in Generative Model and Image Synthesis

Recently, significant success has been achieved by generative models, which aim to mimic humanity’s ability to yield various modalities. The capacity of GPT-series [23,24], Llama series [25,26], Qwen series [27,28] and DeepSeek series [29,30] in the Natural Language Processing field has significantly impacted human’s daily life. In the meantime, stable-diffusion [31,32] and DALL-E [33,34] in computer vision have been proposed for generating high-quality realistic images.

While earlier methodologies like Generative Adversarial Network (GAN)-based methods [35,36,37,38,39,40] and Variational Autoencoder (VAE)-based methods [41,42] demonstrate remarkable performance in yielding realistic samples, training instability remains a well-known issue. For instance, GANs require a delicate balance between the generator and discriminator, which can lead to problems like mode collapse—where the generator produces limited diversity in outputs. This motivated the development of more stable approaches like diffusion models.

Instead of traditional diffusion models (DMs) that denoise the input *x* in the image-scale (pixel level) [43,44], current general text-to-image (T2I) Latent Diffusion Models (LDMs) [31,32,33,45,46] adopt a VAE-like Encoder E and Decoder D structure. LDMs first compress the input into a latent representation z=E(x), and afterwards deploy the diffusion process within the latent space, such that the decoder output $\tilde{x}$ is the reconstructed input *x*. With the hallmark of achieving a favorable trade-off between reducing computational and memory costs while maintaining high-resolution and quality synthesis, operating on smaller spatial latent representations of the input has become a popular framework for recent generative models [47,48,49]. Based on the development of DM-based image generation, some studies also focus on aerial image synthesis [6,48,50,51], but they all concentrate on the common weather rather than multiple domain data.

Beyond T2I image generation, Image-to-Image (I2I) Style Transfer [36,52,53,54,55,56,57,58,59] is a practical generative task that aims to extract style and texture information from one reference image and transfer it to another image while preserving semantic content. Prior methods can synthesize vivid and diverse results, such as converting a landscape photo into a painterly oil artwork. However, for de facto domain shifts in aerial imagery, the performance of these methods is limited for the following reasons: (1) Lack of diverse style references: These methods lack style reference imagery for various domains and a unified environment that provides diverse weather and illumination conditions; (2) Inadequate handling of complex domain shifts: While traditional neural network-based style transfer models [36,52,53,54] can handle atmospheric and illumination changes, they fail to tackle complex domain transfers such as snowy conditions, where physical snow/winter-related elements should be added to the scene, e.g., snow accumulation on rooftops and leafless trees; (3) Content alteration issues: Diffusion model (DM)-based methods [55,56,57,58,59] are prone to altering the original semantic content of images, such as shifts in object positions or deformations of large structures. While such alterations are acceptable in human face style transfer or art editing, preserving geo-spatial information is vital for remote sensing analysis. Moreover, such content alterations render the original semantic segmentation masks unusable, resulting in an additional expensive annotation burden.

ControlNet [45] has recently become a promising approach with the capability to control stable diffusion through various conditioning inputs such as Canny edges, skeletons, and segmentation masks. However, it requires detailed text prompts to achieve consistent target generation in the remote sensing domain. Therefore, in this work, in addition to leveraging segmentation maps as layout conditions, we also utilize them as input for LLM-assisted text descriptor generation. Specifically, for each aerial image’s corresponding segmentation mask, we calculate the pixel ratio for each class and assign each class to one of three levels, *high*, *medium*, or *low*, then construct a scene elements array as input to the LLM. With this approach, detailed and closely scene-corresponding text prompts are generated.

Though a variety of image generation or style transfer models have been developed recently, they are still inadequate and encounter some specific problems on the way to handling domain shift in aerial image processing. However, generative models tend to specialize in a particular task and is equipped with its own merits, e.g., style transfer models can easily change the image scene illumination and atmospheric conditions with a fast inference speed; DM-based methods can greatly edit the images’ content while costing a sequence of sampling steps. Therefore, there is a critical need to adopt multiple models and leverage their advantages to generate diverse weather conditions for aerial imagery while preserving semantic content and geo-spatial information. Recently, Large Language Models (LLMs) [23,24,25,26,27,28,29,30] have emerged as powerful agents capable of orchestrating complex and multi-step tasks. Several pioneering works [60,61,62,63,64] have demonstrated that LLMs can effectively learn to coordinate and utilize diverse tools across multiple modalities and domains, achieving remarkable performance in language processing, computer vision, and other challenging applications. Leveraging LLMs as intelligent agents to automatically select and coordinate appropriate models for addressing diverse domain shift scenarios represents a promising and scalable solution.

### 1.3. Essence and Contributions of This Paper

To address the underestimated domain shift challenge in current remote sensing analysis, particularly in aerial image segmentation, we propose Multi-Weather DomainShifter to overcome the limitation of limited domain variety while eliminating the need for additional annotations on domain-shifted data. Specifically, for multi-weather scene transfer in aerial imagery, given a user’s text input, a LLM agent (e.g., Claude 3.7 Sonnet, GPT-4, DeepSeek R-1, etc.) decomposes the task into simpler steps and systematically plans the procedure for resource identification, appropriate generative tool selection, self-correction, and verification. This paper comprises the following key components:

**Aerial Weather Synthetic Dataset (AWSD):** To complement existing datasets and address their limitations, we developed Aerial Weather Synthetic Dataset (AWSD), which introduces controlled variations in weather and lighting based on Unreal Engine [65]. This dataset provides an ideal benchmark for evaluating the robustness of segmentation models in diverse environmental conditions. Leveraging this dataset, we generated realistic domain-shifted data, which supplements existing aerial image segmentation datasets like ISPRS datasets [10,11]. We specifically focused on overcast, foggy, and dusty weather conditions, which are typical domain shift scenarios that change illumination and introduce atmospheric obscuration elements. This allowed us to demonstrate the effects of domain shift and present domain adaptation results.

**Latent Aerial Style Transfer model (LAST):** Based on the AWSD, we present a latent style transfer model for aerial images. This model transfers domain information from synthetic data while preserving the exact spatial layout and semantic content. In particular, we first utilize a VAE encoder to simultaneously compress both the style reference image and the semantic content image into latent space. The interaction between the style and content is then processed through transformer blocks in this latent space. Finally, the transformed output is decoded back into the image space using the VAE decoder. We transfer clear weather aerial images from the original ISPRS dataset into overcast, foggy, and dusty weather conditions with high computational efficiency (9.45 FPS on a single RTX 4090).

**Multi-Modal Snowy Scene Diffusion Model (MSDM):** In addition to changing illumination and atmospheric information, diffusion models are more appropriate for generating physical element (object)-based scenes such as snowy scenes, e.g., snow-covered roofs and ground. To achieve consistency in image content (including targets of interest and layout), we propose a Multi-Modal Snowy Scene Diffusion Model by leveraging both image conditions and text conditions. Specifically, real aerial images’ segmentation masks are simultaneously served as image conditions controlled by ControlNet [45] and as initial scene descriptions that provide object information in the images. Then, the object information is extended into detailed text prompts by a local-implemented Qwen3-14B [28] model. This approach ensures that original geo-spatial annotations remain valid after transformation, eliminating the need for costly re-annotation.

Beyond these technical contributions, **Multi-Weather DomainShifter** offers significant practical advantages for real-world deployment. Unlike traditional approaches requiring separate preprocessing modules (de-fogging, de-raining, de-snowing) that add inference overhead, this paper provide a intuitive and effective methodology to train recognition models to be inherently robust across all weather conditions without additional runtime modules, which is crucial for time-critical geo-spatial applications like disaster response and environment surveillance. Despite computational requirements during training, the resulting models can be deployed on consumer-grade GPUs with only 6GB VRAM, and our MCP server architecture enables scalable deployment from single-drone operations to city-wide monitoring networks.

Based on the above contributions, we handle the scarcity of domain-specific data in aerial image segmentation. Moreover, we benchmark nine different state-of-the-art segmentation models on multi-domain datasets generated by **Multi-Weather DomainShifter**. Extensive experiments reveal the performance degradation caused by domain shifts, and we successfully adapted model performance in the shifted domain while maintaining its effectiveness in the source domain, providing not just a research contribution but a deployable solution for improving real-world applicability of aerial segmentation models across diverse environmental conditions.

## 2. Related Work

### 2.1. Semantic Segmentation

Following the pioneer approach, i.e., Fully Convolutional Network (FCN) [19], Encoder-Decoder structure has been a prevalent paradigm for semantic segmentation tasks. In the early stage, these methods [21,66,67,68] combined low-level features and up-sampled high levels to obtain precise objects boundaries while capturing global information. Consequently, deeplab-series methods [16,69,70] developed the dilated convolutions to enlarge the receptive field of convolutional layers and further employed spatial pyramid pooling modules to obtain multi-level aggregated feature.

In addition to CNN-based semantic segmentation methods, vision Transformer-based approaches [12,14,17,71] have also become popular due to their exceptional ability to capture long-range contextual information among tokens or embeddings. SETR [72] employs ViT as its backbone and utilizes a CNN decoder to frame semantic segmentation as a sequence-to-sequence task. Moreover, Segmentor [20] introduces a point-wise linear layer following the ViT backbone to generate patch-level class logits. Additionally, SegFormer [73] proposed a novel hierarchically structured Transformer encoder which outputs multiscale features and a MLP decoder to combine both local and global information. Notably, many recent Feature Pyramid Network (FPN) [74]-based affinity learning methods [4,5,15,75] have been proposed to achieve better feature representation and successfully handle the scale-variation problem [22,76] in aerial image segmentation.

### 2.2. Image Style Transfer

Image style transfer [36,52,53,77] is a practical research field that applies the style of one reference image to the content of another. Image style transfer aims to generate a transferred image that contains the content, such as shapes, structures, and objects, of the original content image but adopts the style, such as colors, textures, and patterns of the reference style image. The pioneer method [52] demonstrates that the hierarchical layers of CNNs can extract content and style information, proposing an optimization-based approach for iterative stylization. However, such optimization-based networks are often limited to a fixed set of styles and cannot adapt to arbitrary new ones. To address this limitation, AdaIN [78] presents a novel adaptive instance normalization (AdaIN) layer that aligns the mean and variance of the content features with those of the style features. Later work by Chen et al. [79] employs an internal–external learning scheme with two types of contrastive loss, enabling the generated image to be more visually plausible and harmonious. StyTr^2^ [54] aims to keep the content consistency on art style transfer with a content-aware positional encoding (CAPE) transformer, which increases the computation cost and reduces the inference speed, making it less suitable for high-resolution remote-sensing applications.

Recently, with the great generative capability of latent diffusion models (LDM) [31,32,33,45,46], style transfer methods based on LDM have achieved tremendous progress [55,56,57,58,59]. However, except for DM’s inherent deficiency, i.e., low generation efficiency caused by the multi-step diffusion process, these methods cannot keep the precise layout of the original content image. Recently, LoRA-based [80] techniques [81,82,83,84,85] have shown remarkable efficacy in capturing style from a single image. In particular, B-LoRA [84] and ConsistLoRA [85] fine-tune two attention layers of up-sampling blocks in SDXL [32] to separately control content and style. However, for each reference image and content image pair, they [84,85] need extra LoRA training, which is inefficient for large-scale aerial image processing.

### 2.3. Domain Shift

Domain shift [86] is a well-known challenge that results in unforeseen performance degradation when a model encounters conditions different from those in its training phase. To address this issue, domain generalization (DG) algorithms [87,88,89,90] have been developed to generalize a model across weather conditions and environments unseen during training, where target domain data is unavailable. Additionally, as a sub-field of transfer learning, domain adaptation (DA) methods [91,92,93] have been proposed to adapt a model trained on a source domain to perform effectively on a target domain. Generally, DA algorithms aim to learn a model from labeled source data that generalizes to a target domain by minimizing the distribution gap between the two domains.

The practical application of domain shift solutions is often hampered by the availability of target domain data, which can be rare and difficult to acquire, especially for diverse weather conditions. Moreover, annotating data for new domains is a laborious and time-consuming task. Therefore, unlike methods that rely on real-world target data, our approach utilizes Unreal Engine [65] to build a synthetic dataset encompassing a wide variety of weather conditions (details in Section 4.4). Furthermore, we apply style transfer to augment the existing, finely annotated ISPRS [10,11]. As a result, by performing joint training on both the source and the synthetically shifted domains, our method can effectively mitigate the domain shift problem and its associated performance degradation.

## 3. Methodology

### 3.1. Multi-Weather DomainShifter

**Figure 3:**
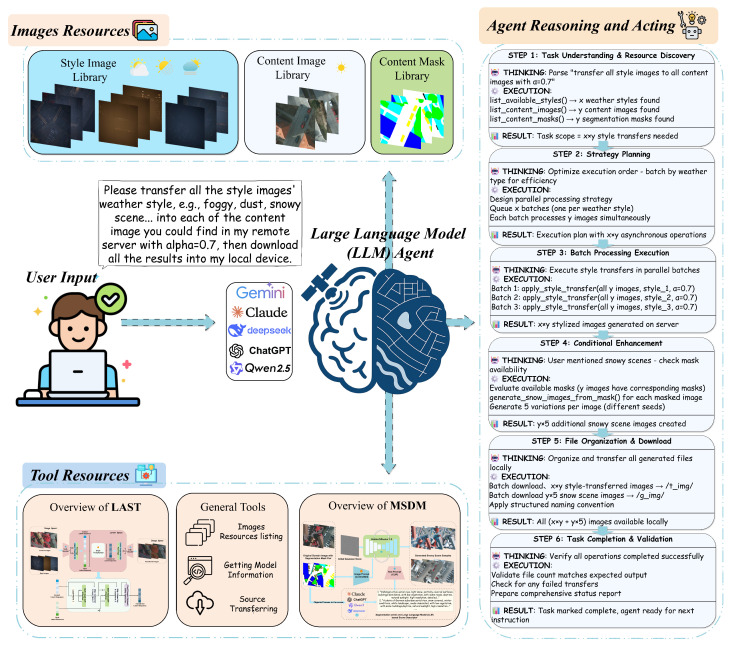
Overview of **Multi-Weather DomainShifter**. The system integrates Image Resources (AWSD style images, content images, and content masks) and Tool Resources (LAST, MSDM, and general tools). Given user text input, an LLM agent performs reasoning and planning to decompose complex domain transfer tasks into executable steps, automatically selecting appropriate tools based on weather conditions and content requirements. (High-resolution figure, zoom in for a better view).

**Multi-weather DomainShifter** is our proposed comprehensive multi-weather domain transfer system that orchestrates multiple generative models to handle diverse weather change-caused domain shift scenarios in aerial imagery. As shown in Figure 3, the system integrates both data resources and specialized tools, coordinated by a Reasoning and Acting (ReAct) framework [94]-based LLM agent [23,24,25,26,27,28,29,30] that can interpret and execute complex, multi-step user commands delivered in natural language.

#### 3.1.1. System Architecture

The architecture of **Multi-Weather DomainShifter** consists of the following three core components:

- *Image Resources:* This component serves as the data foundation for all operations. It is subdivided into three libraries: (1) a Style Image Library containing the target domain style references from our synthetic **AWSD** dataset (e.g., overcast, foggy, dusty), detailed in Section 4.4; (2) a Content Image Library storing the source domain images from real-world datasets like ISPRS [10,11]; and (3) a Content Mask Library with the corresponding semantic segmentation masks for the content images. The samples of style references, original content images, and corresponding segmentation masks are demonstrated in the top part of Figure 3.
- *Tool Resources:* As shown in the bottom part of Figure 3, this is a curated library of specialized generative models and general-purpose utilities. All functions in this tool resources are abstracted as *tools* with descriptions, enabling the LLM agent to understand how they should be utilized. The primary generative tools are our proposed (1) **LAST** model, designed for efficient style transfer of illumination and atmospheric changes (overcast, foggy, dusty), details in Section 3.2; and (2) the **MSDM**, a multi-modal diffusion model for handling complex physical scene alterations like snowy conditions, details in Section 3.3. The library is augmented with general tools for tasks such as resource listing and data transferring.
- *LLM Agent (ReAct Framework):* The system’s intelligence is orchestrated by an LLM agent operating on the ReAct paradigm [94]. This agent synergistically combines reasoning and acting to process user needs, which is illustrated in Figure 3. For each step, it generates a thought process (reasoning), devises an action to execute, and then observes the outcome of that action. This iterative cycle of Thought → Action → Observation allows the agent to dynamically plan, execute, and self-correct until the user’s goal is fully accomplished.

#### 3.1.2. Agent Workflow

The ReAct-based LLM agent follows a conceptual framework that enables autonomous task decomposition and execution, as illustrated in Figure 3. The agent’s workflow operates through several key phases: *Task Understanding*, where natural language instructions are parsed and resource requirements are identified; *Strategic Planning*, where the agent devises optimal execution strategies considering computational efficiency and resource availability; *Tool Selection and Execution*, where appropriate generative models are selected and invoked based on the specific domain transfer requirements; and *Quality Assurance*, where the agent validates outputs and ensures task completion.

This iterative reasoning–acting cycle enables the agent to handle complex, multi-modal domain transfer scenarios that would traditionally require manual intervention. The agent’s ability to dynamically select between **LAST** for atmospheric changes and **MSDM** for physical alterations while coordinating batch processing and resource management demonstrates the system’s capacity for intelligent orchestration of heterogeneous generative models. This architecture ensures that **Multi-Weather DomainShifter** can adapt to diverse user requirements and scale efficiently across different domain shift scenarios.

### 3.2. LAST

**Figure 4:**
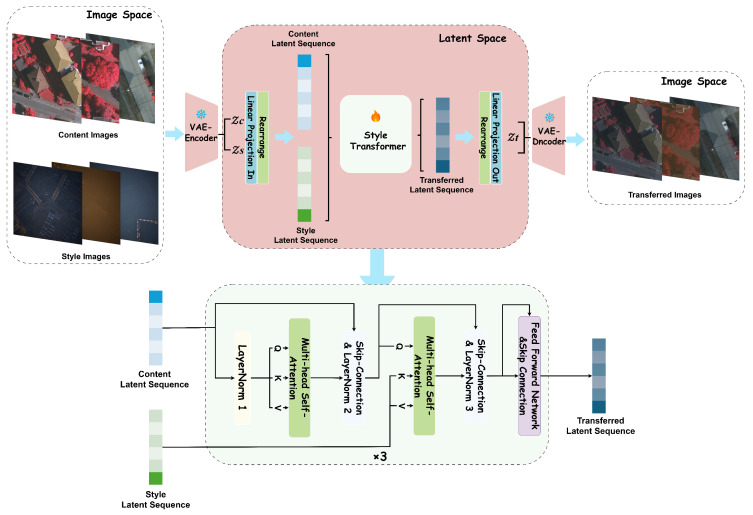
The pipeline of the proposed LAST model. The process takes a pair of content and style images as input. (High-resolution figure, zoom in for a better view).

To achieve style transfer for aerial images, accounting for variations in weather conditions and illumination while reducing the computational cost of processing, we propose the **L**atent-space **A**erial **S**tyle **T**ransfer (**LAST**) model. This model operates in two spaces: image space and latent space, as depicted in Figure 4. Specifically, inspired by the Latent Diffusion Models (LDMs) [31], we first compress the input aerial images into the latent space using a pre-trained VAE. The style transformation is then performed in this latent space.

Overall, this model consists of the following parts: (1) A VAE encoder first compresses both images into a latent space. (2) The resulting latent representations are then flattened into sequences. (3) The core style transfer operation is performed by a latent style transformer that processes these sequences. (4) Finally, a VAE decoder reconstructs the modified latent representation back into the image space, producing the final stylized image. Additionally, the perceptual loss [77], computed via a pre-trained VGG-19 [95], is applied to optimize the model.

#### 3.2.1. VAE for Image Compression

We first deploy the same setup as Latent Diffusion Models (LDMs) [31] to compress images into the latent space via a variational autoencoder (VAE [41,42]) pre-trained with a Kullback–Leibler (KL) divergence penalty.

Given an image $x\in R^{H\times W\times 3}$ in the image space, the encoder E maps *x* to a latent representation $z\in R^{h\times w\times C}$, where h=H/f, w=W/f with a down-sampling factor f=4. Subsequently, the decoder D reconstructs the image $\tilde{x}=D\left(z\right)$ from the latent vector *z*. Specifically, the process within **LAST** involves three steps:

(1) As illustrated in the top-left part of Figure 3, an encoder E maps the input content image $x_{c}$ and style image $x_{s}$ to two separate Gaussian distributions:

(1) $$\begin{array}{cc} N(\mu_{c},\sigma_{c}^{2}) & =E(x_{c}), \end{array}$$

(2) $$\begin{array}{cc} N(\mu_{s},\sigma_{s}^{2}) & =E(x_{s}). \end{array}$$

The reparameterization trick [41,96] is applied to sample the latent vectors $z_{c}$ and $z_{s}$ from their respective distributions:

(3) $$\begin{array}{cc} z_{c} & =\mu_{c}+\sigma_{c}⊙\epsilon , \end{array}$$

(4) $$\begin{array}{cc} z_{s} & =\mu_{s}+\sigma_{s}⊙\epsilon , \end{array}$$

where ⊙ denotes element-wise multiplication, ϵ∼N(0,1) is a noise tensor, and both $z_{c},\;z_{s}\in R^{h\times w\times C}$.

(2) Within the latent space, the vectors $z_{c}$ and $z_{s}$ are processed by the Latent Style Transformer (LSTrans), as shown in the bottom part of Figure 3, which outputs a new latent vector $z_{t}$:

(5) $$z_{t}=\mathrm{LSTrans}\left(z_{c},z_{s}\right),$$

where $z_{t}\in R^{h\times w\times C}$.

(3) Finally, the VAE decoder D reconstructs the stylized image $x_{t}=D\left(z_{t}\right)$, where $x_{t}\in R^{H\times W\times 3}$, as indicated in top-right part of Figure 3.

#### 3.2.2. Latent Style Transformer

The latent representations, denoted as $z\in R^{h\times w\times C}$, are first flattened and embedded into latent sequences, represented as $s\in R^{h\times w\times C}$. To transfer domain-specific information from the input style image to the content image while preserving original semantic details—such as objects, boundaries, and spatial relationships—we stack three sequential transformer blocks in the latent space to process the compressed latent representations. Each block consists of the following components: A Multi-head Self-Attention (MSA) module to grasp contextual information within the content features; A Multi-head Cross-Attention (MCA) module to facilitate interaction between the content and style sequences; A Feed-Forward Network (FFN) to enhance the model’s capacity for non-linear transformation and feature combination.

As a result, **LSTrans** outputs the transferred latent sequence. After being rearranged and projected back to the spatial domain, we obtain the transferred latent representation $z_{t}\in R^{h\times w\times C}$, which is then decoded into the final image $x_{t}\in R^{H\times W\times 3}$ in the image space.

#### 3.2.3. Perceptual Loss for Model Optimization

To guide the model to generate a transferred image $x_{t}$ that preserves the content of $x_{c}$ while incorporating the style of $x_{s}$, we follow established style transfer approaches [54,77,78,79] and employ a perceptual loss (also known as VGG loss). The total loss $L_{\mathrm{total}}$ is a weighted sum of a content loss and a style loss:

(6) $$L_{\mathrm{total}}=\lambda_{c}L_{c}+\lambda_{s}L_{s}$$

where $L_{c}$ computes the content discrepancy between $x_{t}$ and $x_{c}$, and $L_{s}$ computes the style discrepancy between $x_{t}$ and $x_{s}$. The weights $\lambda_{c}$ and $\lambda_{s}$ balance these two components.

Given the pre-trained VGG-19 network and input image $x\in R^{H\times W\times 3}$, we extract features at different depths to capture distinct visual characteristics. The first four convolutional layers output low-level features $f_{l}\left(x\right)$ that encode style and domain information, while the last two convolutional layers output high-level features $f_{h}\left(x\right)$ that encode semantic content. The content loss and style loss are computed as follows:

(7) $$L_{c}={∥f_{h}\left(x_{t}\right)-f_{h}\left(x_{c}\right)∥}_{2}^{2}$$

(8) $$L_{s}={∥f_{l}\left(x_{t}\right)-f_{l}\left(x_{s}\right)∥}_{2}^{2}$$

where $f_{h}\left(x_{t}\right)$ and $f_{h}\left(x_{c}\right)$ represent the high-level features of the transferred image $x_{t}$ and content image $x_{c}$, respectively, while $f_{l}\left(x_{t}\right)$ and $f_{l}\left(x_{s}\right)$ represent the low-level features of the transferred image $x_{t}$ and style image $x_{s}$, respectively.

### 3.3. MSDM

**Figure 5:**
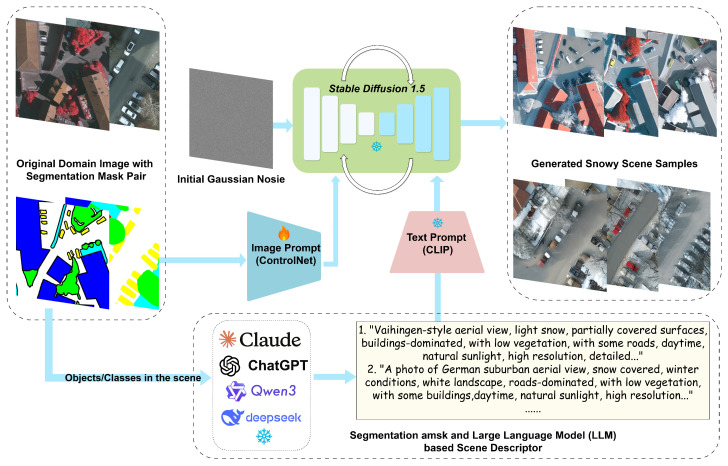
Pipeline of the Multi-Modal Snowy Scene Diffusion Model (MSDM). The system combines segmentation masks through ControlNet with LLM-generated text descriptions to produce snowy aerial scenes maintaining semantic consistency. (High-resolution figure, zoom in for a better view).

To address the challenge of generating realistic snowy aerial scenes while maintaining semantic consistency, we propose the **M**ulti-modal **S**: nowy Scene **D**iffusion **M**odel (**MSDM**). This model integrates ControlNet for structural conditioning with an LLM-assisted scene descriptor to generate contextually rich textual prompts. As illustrated in Figure 5, MSDM ensures that generated snowy scenes preserve the spatial layout and semantic content of the original imagery while incorporating realistic weather-specific visual effects.

#### 3.3.1. ControlNet for Segmentation Mask Conditioning Diffusion Model

As shown in the **Image Prompt** of Figure 5, we employ ControlNet [45] for snowy scene generation to maintain structural consistency between original domain and generated snowy images. ControlNet extends pre-trained diffusion models by introducing additional conditional inputs without requiring complete retraining of the base model [31]. We create a trainable duplicate of the U-Net [66] encoder blocks that processes spatial conditioning information while keeping the original model parameters frozen. Given a segmentation mask $c\in R^{H\times W\times 3}$ and noisy latent $z_{t}$ at timestep *t* obtained by applying the forward diffusion process [31,44,97] to the clean latent representation $z_{0}$, ControlNet generates additional spatial features:

(9) $$z_{t}=\sqrt{{\bar{\alpha }}_{t}}z_{0}+\sqrt{1-{\bar{\alpha }}_{t}}\epsilon$$

(10) $$F_{down},F_{mid}=\mathrm{ControlNet}\left(z_{t},t,e_{text},c\right)$$

where ϵ∼N(0,I) is the ground truth noise and ${\bar{\alpha }}_{t}$ the cumulative noise schedule parameter. $e_{text}$ represents the CLIP [98] text embedding of the input prompt (shared with the original U-Net), $F_{down}$ are the down-sampling block residuals, and $F_{mid}$ is the middle block residual feature. The ControlNet features are integrated into the U-Net prediction through additive residual connections via zero-initialized convolution layers:

(11) $$\epsilon_{\theta }=\mathrm{UNet}\left(z_{t},t,e_{text},F_{down},F_{mid}\right)$$

Our training objective follows the standard diffusion loss with ControlNet conditioning:

(12) $$L=E_{z_{0},c,\epsilon ,t}\left[∥\epsilon -\epsilon_{\theta }\left(z_{t},t,e_{text},c\right){∥}_{2}^{2}\right]$$

For our application, we utilize segmentation masks from the merged ISPRS Vaihingen dataset as control signals. Each mask contains semantic classes including buildings, roads, trees, low vegetation, vehicles, and clutters, converted to RGB format using pre-defined color mapping. Please refer to original papers on DDPM [44], DDIM [97], StableDiffusion [31] and ControlNet [45] for detailed architecture and mechanisms.

#### 3.3.2. LLM-Assisted Scene Descriptor

To enhance the quality and realism of generated snowy scenes, we incorporate textual descriptions generated by Qwen3-14B [28]. Unlike fixed templates or manual annotations, our method performs intelligent scene analysis to generate contextually rich and semantically accurate prompts. As indicated in the bottom part of Figure 5, the LLM analyzes the segmentation masks and generates detailed scene descriptions that capture the semantic content, which are then used as additional conditioning information for the diffusion model.

**Segmentation Mask Analysis**. The LLM-assisted descriptor begins with quantitative analysis of the segmentation mask. For each semantic class *k*, we compute the pixel ratio:

(13) $$r_{k}=\frac{\sum_{i,j}1\left[m\left(i,j\right)=k\right]}{H\times W}$$

We assign semantic importance levels based on class-adaptive thresholds:

(14) $${\mathrm{level}}_{k}=\left\{\begin{array}{cc} \mathrm{high} & \mathrm{if}\;r_{k}\geq \tau_{k}^{high}\\ \mathrm{medium} & \mathrm{if}\;\tau_{k}^{medium}\leq r_{k}<\tau_{k}^{high}\\ \mathrm{low} & \mathrm{if}\;\tau_{k}^{low}\leq r_{k}<\tau_{k}^{medium}\\ \mathrm{None} & \mathrm{if}\;r_{k}<\tau_{k}^{low} \end{array}\right.$$

The thresholds $\tau_{k}$ are empirically determined based on typical class distributions in aerial imagery. For instance, vehicles require lower thresholds ($\tau_{vehicle}^{low}=0.01$) due to their smaller spatial footprint, while buildings and roads use higher thresholds ($\tau_{building}^{low}=0.10$).

**Structured Prompt Generation**. We select the top-3 most prominent scene elements based on pixel ratios and construct structured LLM input:

(15) $$S={\left\{{\mathrm{element}}_{i},{\mathrm{level}}_{i},r_{i}\right\}}_{i=1}^{3}$$

where elements are sorted by descending ratio: $r_{1}\geq r_{2}\geq r_{3}$. The LLM input combines scene context with quantitative analysis:

(16) Input={city_type : c,weather : w,time : t,scene_elements : S}

where *c* represents the urban/suburban classification, *w* specifies the target weather condition (snowy), and *t* indicates the temporal context (day/night). The LLM generates structured textual descriptions that serve as conditioning prompts for ControlNet training:

(17) prompt=LLM(Input)=Qwen3-14B(c,w,t,S)

This generated prompt is subsequently encoded by the CLIP text encoder to produce the text embeddings $e_{text}$ used in Equations (10)–(12).

## 4. Experiments

Existing aerial image segmentation datasets, such as ISPRS Vaihingen and Potsdam [10,11], serve as widely used benchmarks, offering high-resolution, annotated images of urban environments. While these datasets are invaluable for training and evaluating segmentation models, they have significant limitations in real-world applications. A key issue is the lack of diversity in environmental conditions. As a result, it does not accurately reflect the variability present in real-world aerial imagery [5]. Consequently, models trained on these datasets often struggle with domain shifts—environmental changes like weather or lighting variations that can drastically reduce segmentation accuracy.

In real-world scenarios, such as disaster response or urban planning, aerial images are frequently taken under challenging conditions, including overcast, fog, and snow. The absence of such environmental diversity in standard datasets limits the robustness and adaptability of segmentation models when deployed in dynamic environments. To address this shortcoming, there is a need for a new dataset that not only mirrors the spatial characteristics of datasets like ISPRS but also includes diverse weather conditions to simulate domain shifts.

The experimental evaluation in this section is organized as follows: We first introduce the ISPRS Vaihingen and Potsdam datasets [10,11] in Section 4.1. Section 4.2 presents the implementation details and computational requirements of our LAST/MSDM models. In Section 4.3, we demonstrate the weather change-caused domain shift effects on model performances using the Vaihingen dataset. Section 4.4 introduces our proposed AWSD dataset. In Section 4.5, we conduct an ablation study to verify our generated data effectiveness and generalization capability, including intra-distribution experiments and cross-distribution experiments. Finally, in Section 4.6, we present a comprehensive study to demonstrate the domain adaptation effects.

### 4.1. ISPRS Dataset

The International Society for Photogrammetry and Remote Sensing (ISPRS) Vaihingen dataset and Potsdam datasets [10,11] are widely used benchmarks from the ISPRS 2D Semantic Labeling Contest.

The Vaihingen dataset consists of high-resolution true orthophotos of Vaihingen, Germany, with a ground sampling distance (GSD) of 9 cm. It includes 33 image tiles, 16 of which are annotated with six semantic categories: *impervious surfaces, buildings, low vegetation, trees, cars, and clutter (background)*. The Potsdam dataset offers a finer GSD of 5 cm, containing 38 tiles of diverse urban scenes with the same six-class annotation scheme. Specifically, the original high-resolution images were processed into non-overlapping 512 × 512 patches. The resulting Vaihingen dataset contains 344 patches for training and 398 for validation and Potsdam dataset contains 3456 patches for training and 2016 for validation.

For our experiments, we mainly use Vaihingen dataset for numeric comparison, including domain shift effect demonstration (details in Section 4.3) and final comparison study (details in Section 4.6). Meanwhile, we use both the Vaihingen and Potsdam datasets for the capacity verification (details in Section 4.5) of synthetic data generated by **LAST** and **MSDM**.

### 4.2. Model Implementation Details

We introduce the detailed model training configurations in Section 4.2.1, Section 4.2.2 and Section 4.2.3. In addition, Table 1 summarizes the training time and resource consumption for each model. Moreover, we demonstrate the computational cost of each component of the system to show its efficiency in Section 4.2.4, where Table 2 shows the detailed inference cost for all the models in this paper and Table 3 further estimates the models’ performance on handling large-scale data.

#### 4.2.1. Detailed Setup of LAST

The LAST model (introduced in Section 3.2) uses both source-domain content images and target-domain style references. We use ISPRS Vaihingen as the source-domain content images, while the target-domain style is derived from the 1386 synthetic images from our AWSD dataset (462 images for each weather condition: *overcast*, *foggy*, *dusty*), which were generated using Unreal Engine 5 [65].

The latent style transformer (Section 3.2.2) is trained for 160,000 iterations on two NVIDIA RTX 4090 24 GB GPUs using the Adam optimizer [99] with learning rate of 5 × 10^−4^ and learning rate decay of 1 × 10^−5^. The batch size is set to 8. To preserve their pre-trained representations, the parameters of both the VAE (Section 3.2.2) and the perceptual VGG-19 feature extractor (Section 3.2.1) remain frozen throughout the training process.

Moreover, during DomainShifter’s *Tool Selection and Execution* process in Section 3.1.2, we use a strength parameter α∈[0,1] in post-processing that controls style intensity through linear interpolation between the transferred/stylized output and the original content image. This process can be formally described as follows:

(18) $$x_{final}=\alpha \cdot x_{t}+\left(1-\alpha \right)\cdot x_{c}$$

where $x_{final}$ represents the final output image, $x_{t}$ is the transferred image from **LAST**, and $x_{c}$ is the original content image (see Section 3.2).

**Table 1:** Model architecture and training configuration.

Model	Parameters (M)	Trainable (M)	Batch Size	Time (hours)	Iters/Epochs
*Generative Models:*					
LAST	128.5	31.9 (24.8%)	8	48	160K iters
MSDM	1427.5	361.3 (25.2%)	32	12	50 epochs
*ResNet-50-Based Models:*					
PointRend-R50 [18]	28.7	28.7 (100.0%)	24	7.8	80K iters
DeepLabV3+-R50 [16]	43.6	43.6 (100.0%)	24	21.4	80K iters
PSPNet-R50 [21]	49.0	49.0 (100.0%)	24	20.4	80K iters
FCN-R50 [19]	49.5	49.5 (100.0%)	24	16.4	80K iters
DANet-R50 [17]	49.8	49.8 (100.0%)	24	18.7	80K iters
UperNet-R50 [15]	66.4	66.4 (100.0%)	24	16.2	80K iters
*Transformer-Based Models:*					
UperNet-Swin	59.8	59.8 (100.0%)	24	18.7	80K iters
UperNet-ViT	144.1	144.1 (100.0%)	20	20.4	80K iters
Segmenter-ViT [20]	102.4	102.4 (100.0%)	1	1.0	80K iters

#### 4.2.2. Detailed Setup of MSDM

The MSDM approach (details in Section 3.3) leverages both visual and textual information as the generation condition. We utilize ISPRS Vaihingen as the source domain image set along with its corresponding segmentation masks as image input. Subsequently, based on these segmentation masks, LLM-assisted scene descriptors generate the corresponding text input. We implement the training pipeline with *Accelerate* for distributed training support. The ControlNet model is initialized from the pre-trained segmentation ControlNet checkpoint from *Huggingface* and then fine-tuned on our weather-augmented dataset.

The base model uses Stable Diffusion v1.5 [31]. To preserve pre-trained knowledge, the VAE Encoder/Decoder, U-Net, and CLIP text encoder parameters are frozen, while only ControlNet parameters are optimized, significantly reducing memory requirements. We employ standard MSE loss between predicted and ground truth noise in the latent space. For optimization, we employ 4 × RTX5090 32 GB GPUs with AdamW optimizer [100] (learning rate: 1 × 10^−5^, weight decay: 0.01, constant warm-up scheduler). The LLM used in Section 3.3.2 operates with temperature T=0.7 and a 200-token limit, producing 70–100 word prompts optimized for diffusion model performance. During inference, the model is implemented on two RTX 4090 24 GB GPUs with device mapping set as *auto*. Generation uses DDIM [97] sampling with 30 steps and guidance scale 7.5. Finally, we generate 5 different sets of snowy scene data with random seeds of {46, 50, 51, 53, 54}.

#### 4.2.3. Detailed Setup of Semantic Segmentation Models

We implement all the segmentation models under supervised training and testing based on the MMSegmentation [101] toolbox for fair and comprehensive segmentation, including UperNet [15] with three different backbones (Swin Transformer [12], ResNet-50 [13], and ViT-Base [14]), DeepLabV3+ [16], DANet [17], PointRend [18], FCN [19], Segmenter [20], and PSPNet [21], all with a ResNet-50 backbone except where specified. In this section, for simplicity, we only showcase the detailed configuration of DeepLabV3+ with ResNet-50 as a backbone as an example.

In particular, we employ this prevalent segmentation model for its excellent performance and generality on various domain semantic segmentation tasks, including autonomous driving (Cityscapes dataset [102]), common segmentation tasks (COCO dataset [103]), and aerial image segmentation (ISPRS dataset [10]). The detailed training process involves 80,000 iterations on 2 × NVIDIA RTX 4090 24 GB GPUs using SGD optimizer with momentum 0.9 and weight decay 0.0005. The learning rate follows a polynomial decay schedule (PolyLR) starting from 0.01 and decreasing to 0.0001 with power 0.9. The batch size is set to 24, and standard data augmentation techniques including *RandomResize, RandomCrop, RandomFlip, and PhotoMetricDistortion* are applied.

In the experimental stage, we first obtain the augmented multi-domain dataset generated by **LAST** and **MSDM**, consisting of 3440 training images across five weather conditions: real clear weather (344 images), overcast (344 images), foggy (344 images), dusty (344 images), and snowy scenes with five different variants (2064 images total from random seeds of 46, 50, 51, 53, 54). The validation set contains 398 images from the original ISPRS Vaihingen dataset and the same number of images for each transferred domain, i.e., overcast, foggy, etc.

Here, we must emphasize that for all of the following studies on effect of domain shift in Section 4.3, the ablation study in Section 4.5, and the comprehensive evaluation in Section 4.6), *we train the models using supervised learning*. In particular, for Section 4.3, we train the segmentation models on only 344 clear weather images and validate them on various weather domains; for Section 4.5, we train the segmentation models following the detailed experiment ID 1–7’s setting. For the last comparison experiment in Section 4.6, we leverage all the training data clear weather (344 images), overcast (344 images), foggy (344 images), dusty (344 images), and snowy scenes with five different variants (2064 images) to improve the models’ robustness against weather change-induced domain shift, then we verify the performance on all weather validation sets, respectively.

#### 4.2.4. Inference Cost and Performance

In order to conduct fair and accurate complexity computation, all the models’ inference benchmarks were conducted on an Ubuntu 22.04 64-bit device equipped with one Intel Core i9-14900K CPU and a single NVIDIA RTX 4090 24GB GPU. Moreover, the software environment consists of *CUDA* 12.6, *PyTorch* 2.7.0, *Torchvision* 0.22.0, and *Transformers* 4.52.3. The input is with FP32 precision and 512×512 input resolution. Notably, we employ *thop* [104] to calculate the precise FLOPs. The details are illustrated in Table 2. This table clearly illustrates the hardware requirements for implementing this system. Although all models were trained on different GPU configurations based on their computational and optimization demands (e.g., 2×2 RTX4090 24GB GPUs for **LAST** and 4×4 RTX5090 32GB GPUs for **MSDM**), it is worth noting that all models can be deployed for inference on consumer-grade GPUs with only 6GB of VRAM, making our system accessible for practical applications. This demonstrates that despite the substantial computational requirements during training, the deployment phase is remarkably efficient and hardware-friendly.

The efficiency gap between LAST and MSDM becomes critical for large-scale data augmentation. As shown in Table 2, **MSDM**’s FLOPs and inference time are much higher than those of the **LAST** model. In the meantime, as shown in Table 3, generating 1000 512×512 atmospheric images using LAST requires only 1.8 min, whereas MSDM needs 57 min for snowy scene generation. This efficiency gap clearly demonstrates the inherent computational overhead of diffusion models, since they require 30 DDIM steps for the denoising process. This makes LAST particularly suitable for massive dataset augmentation, while MSDM is strategically deployed for complex physical transformations that justify its computational cost.

**Table 2:** Comprehensive inference performance comparison. All segmentation models measured on 1× NVIDIA RTX 4090 24GB with FP32 precision at 512 × 512 resolution.

Model	FLOPs (TFLOPs)	Inference Time (ms)	FPS (img/s)	VRAM (GB)
*Generative Models:*				
LAST	2.330	105.9	9.45	1.44
MSDM	15.446	3426.9	0.29	4.37
*ResNet-50-Based Models:*				
FCN-R50 [19]	0.198	12.4	80.96	0.40
PSPNet-R50 [21]	0.179	11.8	84.45	0.47
DANet-R50 [17]	0.211	12.6	79.33	0.41
DeepLabV3+-R50 [16]	0.177	12.4	80.85	0.50
UperNet-R50 [15]	0.237	17.2	58.20	0.68
PointRend-R50 [18]	0.034	14.7	68.15	0.30
*Transformer-Based Models:*				
UperNet-Swin	0.236	25.3	39.51	0.64
UperNet-ViT	0.443	22.2	44.99	1.00
Segmenter-ViT [20]	0.126	13.8	72.45	0.51

**Table 3:** Large-scale data processing cost estimation under 1× NVIDIA RTX 4090 24 GB with FP32 precision at 512×512 resolution.

Task	Model	Images	Total Time	GPU Hours
Atmospheric transfer	LAST	1000	1.77 min	0.029 h
Snowy scene generation	MSDM	1000	57.1 min	0.95 h
Segmentation inference	DeepLabV3+	1000	12.4 s	0.0034 h

### 4.3. Effect of Weather Change-Caused Domain Shift

To demonstrate the effect of weather change-caused domain shift, using the original ISPRS training set, we trained the following nine semantic segmentation models: UperNet with three different backbones (Swin Transformer [12], ResNet-50 [13], and ViT-Base [14]), DeepLabV3Plus-ResNet-50, DANet-ResNet-50, PointRend-ResNet-50, FCN-ResNet-50, Segmentor-ViT-Base, and PSPNet-ResNet-50 [15,16,17,18,19,20,21]. We then evaluated their performance on five domains: the original ISPRS validation set and its style-transferred counterparts {*overcast*, *foggy*, *dusty*, *snowy*} generated by **LAST** and **MSDM**. The overview results are illustrated in the radar chart in Figure 2, and detailed numeric results based on mIOU and mFscore are shown in Table 4 and Table 5, respectively.

Table 4 and Table 5 reveal a clear pattern of performance degradation (compared to the original performance under clear weather) as domain shift severity increases. Under **overcast** conditions, where image content remains unchanged but illumination is slightly reduced, all models experience performance drops with average mIoU and mFscore deteriorations of **3.35%** and **2.62%**, respectively. When atmospheric conditions are further compromised in **foggy** and **dusty** scenarios—where both illumination changes and reduced atmospheric visibility occur—more severe domain shift leads to progressively worse performance, with drops of **3.92%**/**3.24%** and **10.55%**/**8.66%** for mIoU/mFscore, respectively. The most dramatic degradation occurs under **snowy** conditions, where scene targets are partially occluded or color-altered (e.g., snow covering rooftops), resulting in substantial performance drops of **28.59%** and **25.76%** for mIoU and mFscore, respectively. These results underscore the critical impact of domain shift on semantic segmentation performance, even when the underlying scene structure remains unchanged.

Notably, our analysis reveals that ViT-based backbones demonstrate superior domain robustness compared to CNN-based alternatives. UperNet-ViT-B exhibits the best resilience under mild weather variations, with minimal drops (**1.00%** mIoU under overcast/foggy conditions), while Segmenter-ViT-B shows the most robust performance under severe conditions (dusty: **4.32%** mIoU drop, snowy: **23.95%** mIoU drop), significantly outperforming ResNet-50-based models, which suffer up to **30.81%** mIoU degradation under snowy conditions.

### 4.4. Synthetic Dataset

**Figure 6:**
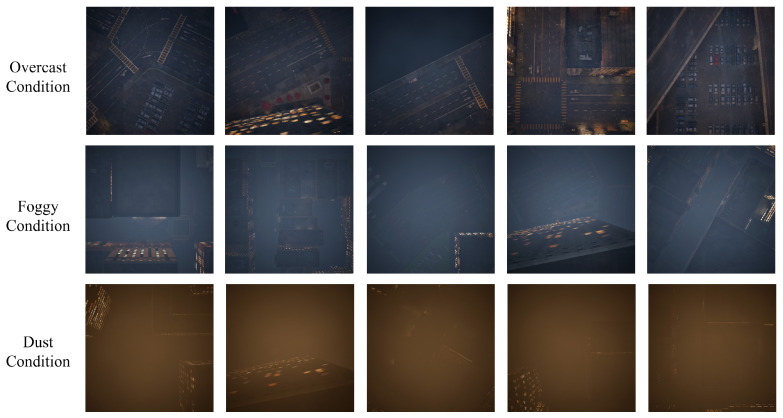
Visual samples from our synthetic **A**erial **W**eather **S**ynthetic **D**ataset (**AWSD**) created with Unreal Engine 5 [65]. Each row showcases a different environmental condition applied to the different urban scenes, providing style references for conditions that are scarce in real-world aerial benchmarks. (High-resolution figure, zoom in for a better view).

To rigorously evaluate model performance under domain shift, we introduce the **A**erial **W**eather **S**ynthetic **D**ataset (**AWSD**), a synthetic dataset created using Unreal Engine 5 [65]. AWSD is designed to replicate realistic urban environments modeled based on the Potsdam and Vaihingen datasets. Images are captured from a 200-m aerial perspective, maintaining consistency with the original benchmarks in terms of viewpoint and object layout. Visual examples of our synthetic data are presented in Figure 6.

In contrast to the static, clear-sky conditions of the ISPRS datasets [10,11], AWSD incorporates a diverse range of environmental variations, including challenging weather conditions as well as different illumination settings. As shown the samples from Figure 6, from top to bottom, we modulate the weather from *overcast*, *foggy* to the *dusty* based on Unreal Engine 5 environment [65]. These scenarios were purposefully introduced to assess the adaptability of segmentation models to significant domain shifts. Crucially, AWSD retains the same pixel-level semantic annotations across the six urban categories as ISPRS, ensuring a fair and precise evaluation for both small and large objects in complex environments.

Therefore, by systematically introducing these varied scenarios, AWSD directly addresses the challenge of domain generalization. Its synthetic nature enables the consistent and controllable simulation of environmental variations that are difficult and costly to capture in real-world data acquisition solutions. This makes AWSD a valuable resource for developing and benchmarking aerial segmentation algorithms with enhanced robustness for real-world applications.

### 4.5. Ablation Study of Synthetic Data Verification

To evaluate the effectiveness and transferable domain adaptation ability of synthetic data generated by **Multi-Weather DomainShifter**, we augment both the original ISPRS Vaihingen training set and validation set with generated four different domain images. Meanwhile, we generate the various domain data for **only** the Potsdam validation set, because Potsdam contains only the original data but not any weather-shifted data.

For simplicity, we take the prevalent Deeplabv3+ [16] model with ResNet-50 as the backbone [13] for the ablation study and conduct the following comprehensive seven experiments, explained below as Exp. 1 to Exp. 7, where the numerical results of Exp. 1 to Exp. 7 are shown in Table 6 and Table 7. Meanwhile, the following abbreviations are used: VN: Vaihingen, Ori: Original, VN Weather (w/o. snow): atmospheric changed data, i.e., overcast, foggy and dusty, VN All Weather (w. snow): All synthetic data in Vaihingen Training set. Notably, for snowy scene generation by the Diffusion Model-based **MSDM**, we average the generated results of the five sets.

**Exp. 1** Train model on only original Vaihingen training set and test on all domains validation set of Vaihingen;

**Exp. 2** Train model on both original Vaihingen training set and **LAST** generated atmospheric changed data, i.e., overcast, foggy and dusty, abbreviated in VN weather (w/o. snow);

**Exp. 3** Train model on all the Vaihingen Domain data, including the generation from **LAST** and 5 different set of snowy scene from **MSDM**, abbreviated in VN ALL Weather (w. snow);

**Exp. 4** Train model on only original Potsdam training set and test on all domains validation set of Potsdam;

**Exp. 5** Train model on both original Potsdam training set and Vaihingen training set;

**Exp. 6** Train model on both original Potsdam training set and the same various domain data in Exp. 2, i.e., VN weather (w/o. snow);

**Exp. 7** Training model on original Potsdam training set and all domains training sets in Exp. 3, i.e., VN ALL Weather (w. snow).

In general, the ablation studies are divided into two main stages. We first conduct **intra-distribution** validation within the same geographic distribution (Exp. 1–3 for Vaihingen Dataset) and **cross-distribution** validation by transferring the weather knowledge from generated data in Vaihingen training set into a new, unseen geographical distribution, i.e., ISPRS Potsdam dataset (Exp. 4–7). For detailed per-class performance breakdown and spread analysis across all semantic categories, please refer to the extended results in Table A1 and Table A2 of Appendix A.

Stage 1: Intra-Distribution Domain Adaptation

The results from Experiments 1–3 demonstrate the effectiveness of synthetic weather data augmentation for enhancing domain adaptation capabilities within the same geographical distribution.

The introduction of atmospheric weather data without snow (Exp. 2) yields consistent performance gains: +3.43% mIoU for overcast, +4.40% mIoU for foggy, and a remarkable +14.31% mIoU improvement for the harsher dusty conditions. Similarly, mFscore improvements of +3.04%, +4.03%, and +12.01% are observed for overcast, foggy, and dusty conditions, respectively. The comprehensive weather augmentation including snow data (Exp. 3) further enhances model robustness, achieving a substantial +19.39% mIoU and +17.44% mFscore improvement in snowy conditions while maintaining competitive performance across other weather scenarios. Comparing the baseline model trained solely on original Vaihingen data (Exp. 1) with the weather-augmented configurations reveals substantial improvements across all atmospheric conditions.

Stage 2: Cross-Distribution Knowledge Transfer

The cross-distribution validation experiments (Exp. 4–7) provide crucial evidence that synthetic data introduces genuine weather-related knowledge rather than causing data leakage artifacts that refers to the potential issue where performance improvements might result from the model simply memorizing training data patterns or exploiting unintended correlations between training and validation sets, rather than learning generalizable weather-related visual features. This stage validates the generalization capability of weather-specific features learned from synthetic data.

A critical observation emerges from Exp. 5, in which adding only original Vaihingen real data to Potsdam training set results in performance degradation for overcast (−2.95% mIoU, −2.51% mFscore) and foggy (−3.99% mIoU, −3.16% mFscore) conditions compared to the Potsdam baseline (Exp. 4). Meanwhile, dusty domain performance shows improvement (+10.19% mIoU, +10.20% mFscore) and snowy performance remains almost unchanged, suggesting that simple dataset combination without weather-specific augmentation provides limited domain adaptation benefits.

The additional atmospheric weather data without snow (Exp. 6) demonstrates the effectiveness of weather-specific knowledge transfer. Performance is restored for overcast and foggy conditions while achieving dramatic improvements in dusty domain (+27.98% mIoU, +26.01% mFscore). Notably, snowy scene performance remains comparable to baseline (+1.23% mIoU, +1.39% mFscore), confirming that without snow-specific training data, the model cannot effectively adapt to snowy conditions through other weather augmentations alone.

The final evaluation (Exp. 7) utilizing all synthetic weather data validates the full potential of the proposed approach. Compared to the Potsdam baseline (Exp. 4), substantial improvements are achieved across all weather conditions: **+2.12%** mIoU and **+1.86%** mFscore for overcast, **+1.72%** mIoU and **+1.50%** mFscore for foggy, **+29.92%** mIoU and **+27.47%** mFscore for dusty, and **+5.87%** mIoU and **+6.34%** mFscore for snowy conditions.

The systematic comparison between Experiments 6 and 7 against both the baseline (Exp. 4) and control group (Exp. 5) provides compelling evidence for the effectiveness and generalization capability of synthetic weather data. The results conclusively verify this paper’s essence that *incorporating additional domain-specific synthetic data significantly enhances model domain adaptation ability and robustness against domain shift*.

Moreover, the successful cross-distribution transfer from Vaihingen to Potsdam particularly demonstrates that synthetic weather knowledge generalizes beyond the original geographical context, indicating that the generated data captures fundamental weather-related visual features rather than dataset-specific artifacts. This generalization capability is essential for practical deployment scenarios where models encounter diverse geographical and environmental conditions not represented in the original training distribution.

### 4.6. Comprehensive Study of Domain Adaptation

**Figure 7:**
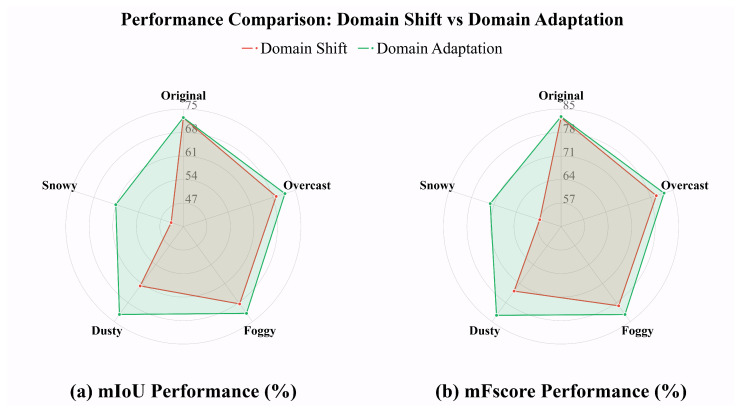
Visualization of domain adaptation recovery (*Green Line*) with generated various domain-specific data compared to domain shift (*Red Line*) on the ISPRS Vaihingen datasets. This comparison study, reports both mIoU (**a**) and mFscore (**b**) metrics. We average the results of 9 prevalent segmentation methods. (High-resolution figure, zoom in for a better view).

**Figure 8:**
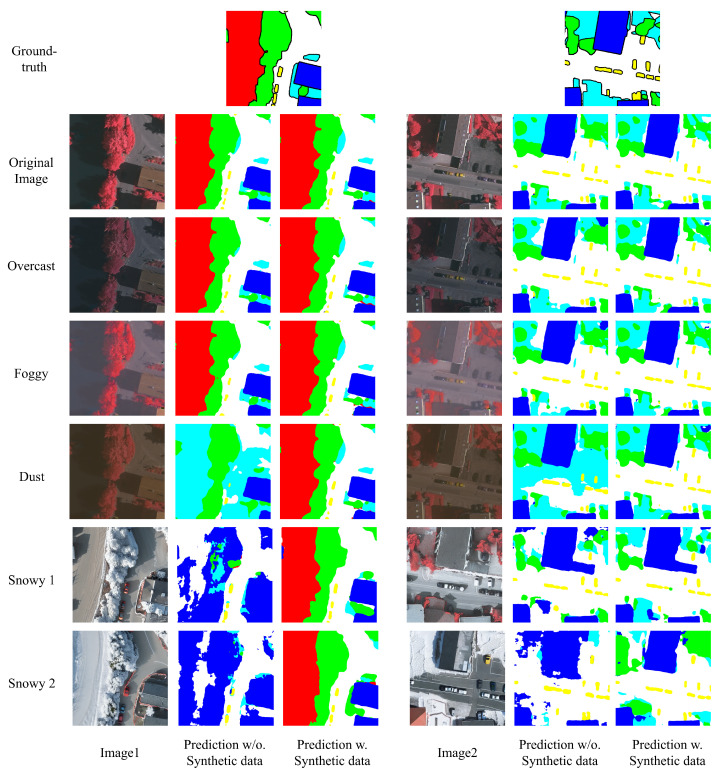
Visualization of predictions results of two set of samples from Vaihingen dataset. Notably, the Snowy1 and Snowy2 are generated from random seed 46 and 51, respectively. (High-resolution figure, zoom in for a better view).

Finally, we employ all the generated domain data to re-train and benchmark all the nine semantic segmentation models on ISPRS Vaihingen dataset [10,11]. The comprehensive results are detailed in Table 8 and Table 9. In addition, for better visualization, the comparison between degradation results caused by domain shift in Table 4, Table 5, Table 8 and Table 9 is shown in the radar chart in Figure 7. Moreover, some samples of prediction are also visualized in Figure 8, where we still take Deeplabv3+ [16] here for simplicity. In particular, we sampled two images from validation set of ISPRS Vaihingen dataset [10,11] and compared their prediction results under diverse weather domain with only original training data (denotes as *Prediction w/o. Synthetic data*, i.e., domain shift results) and with all the synthetic data from {overcast, foggy, dusty and snowy} (denoted as *Prediction w. Synthetic data*, i.e., domain adaptation results).

Our primary evaluation focuses on the robustness and domain adaptation capacity of these models against domain shifts. By retraining on the augmented data, the models exhibit significant performance improvements on the shifted validation sets. As demonstrated in Figure 8, the predictions (i.e., segmentation masks) of Deeplabv3+ without any synthetic data (shown in the column *Prediction w/o. Synthetic data*) gradually deteriorate as the weather conditions progress from easy to difficult. When the weather becomes dusty, where illumination and atmospheric conditions are altered, the shadow regions in *Image1* and *Image2* cannot be correctly handled by the model. Additionally, when the weather shifts to snowy scenes where objects (e.g., the tree in Image1 and buildings in Image2) become white or are covered by snow, the model fails to classify them correctly. In contrast, under the same conditions, after retraining the model with synthetic data from all shifted domains, the model demonstrates clear robustness across all weather conditions (shown in the column *Prediction w. Synthetic data*).

Numerically, compared to the deterioration results in Table 4 and Table 5 (red line in the radar chart in Figure 7), substantial improvements are achieved across all weather conditions: **+2.75%** mIoU and **+2.47%** mFscore for overcast, **+3.48%** mIoU and **+3.20%** mFscore for foggy, **+10.53%** mIoU and **+8.89%** mFscore for dusty, and **+17.55%** mIoU and **+15.74%** mFscore for snowy conditions. The results demonstrate significant performance improvement with the synthetic weather data; meanwhile, the model performances on the original real set remain steady. The comprehensive results demonstrate the effectiveness of our proposals for improving robustness against various domain shifts and enhancing domain adaptation capability.

## 5. Discussion

### 5.1. Comparison with Existing Approaches and Advantages

Compared to existing domain adaptation and style transfer methods, our approach offers several distinct advantages. Traditional domain adaptation methods [91,92,93] require real target domain data, which is expensive to collect and annotate for multiple weather conditions. Style transfer methods [52,54,78] can alter atmospheric appearance but fail to preserve precise spatial layouts—critical for geo-spatial analysis where original annotations must remain valid. Recent diffusion-based methods [55,56,57] achieve impressive visual quality but suffer from content alterations and computational inefficiency for large-scale processing.

Our **Multi-Weather DomainShifter** uniquely addresses these limitations through: (1) **Layout preservation**: Unlike existing style transfer and diffusion methods, our approach maintains exact spatial layouts, enabling reuse of original annotations without costly re-labeling; (2) **Multi-weather handling**: Our system orchestrates specialized models (LAST for atmospheric changes at 9.45 FPS, MSDM for physical alterations) coordinated by an LLM agent, providing comprehensive weather coverage rather than single-condition solutions; (3) **Synthetic-to-real transfer**: Rather than using purely synthetic data, we transfer weather characteristics from controlled synthetic references (AWSD) to real imagery, maintaining real-world statistical properties; (4) **Practical efficiency**: Our trained models require only 6GB VRAM for deployment, with inference times comparable to baseline segmentation models (Table 2), eliminating the need for separate weather-specific preprocessing modules that increase latency.

### 5.2. Real-World Implementation and Practical Significance

While this paper presents two generative models—**LAST** for atmospheric changes and **MSDM** for physical changes such as snowy conditions—our fundamental objective is to address the weather-induced domain shift problem that has been largely overlooked in the remote sensing and aerial image analysis community.

Existing solutions for handling adverse weather conditions, including denoising, de-raining, de-fogging, and de-snowing methods, introduce significant practical challenges in real-world deployment scenarios such as autonomous driving and aerial image segmentation tasks. These additional preprocessing modules impose extra computational costs, hardware burdens, and most critically, increase the computational FLOPs and inference latency. In remote sensing analysis applications, particularly in post-disaster response scenarios where complex weather conditions are common, computational efficiency is paramount. Therefore, our approach fundamentally addresses this problem in an end-to-end manner by generating diverse weather-augmented data to train more robust and generic models.

Our ablation study (Section 4.5) and comprehensive comparison study (Section 4.6) validate the effectiveness, generalizability, and transferability of our methodology. Notably, by maintaining identical segmentation model parameters, our weather-augmented training enables excellent performance across multiple weather domains simultaneously. This eliminates the need for weather-specific preprocessing modules and their associated computational overhead during inference.

It is crucial to emphasize the novelty of our approach compared to existing style transfer and generative models in both academic and industrial contexts. Current style transfer and diffusion-based generative models inevitably alter the spatial layout and content of input images. This limitation poses severe problems for geo-spatial analysis systems, as it renders the original semantic annotations unusable and introduces substantial additional annotation and labor costs. **Our method preserves the original image content and layout**, thereby addressing the critical drawback of previous work. Moreover, unlike approaches that directly employ purely synthetic data for augmentation, our methodology transfers weather-specific styles, features, and characteristics to real aerial data, ensuring that the augmented data maintains the statistical properties and semantic consistency of the original real-world imagery while incorporating realistic weather variations.

### 5.3. Limitations and Unsuccessful Cases

#### 5.3.1. Generative Model Limitations

Due to the probabilistic nature of diffusion models, MSDM occasionally produces failure cases in generating snow-related elements. Although our approach employs an LLM-based description generator to produce rich and accurate text prompts, we do not train the CLIP text encoder. Instead, we train only the U-Net image encoder to align image features with pre-trained text embeddings. To mitigate these failure cases, additional training strategies and more advanced feature alignment mechanisms are necessary.

**Figure 9:**
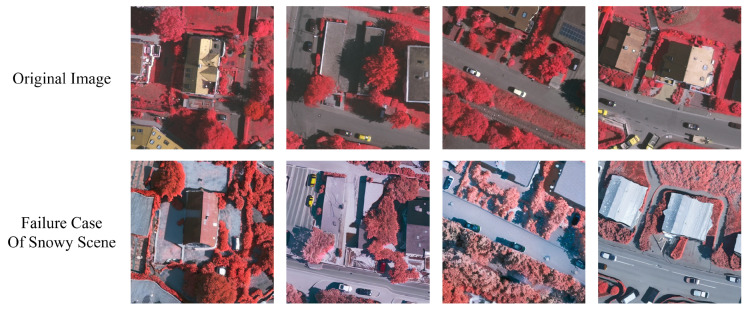
Failure cases of snowy scene generation of the proposed **MSDM**.

Furthermore, to demonstrate the generic applicability of our methodology, we employ only standard supervised learning in our experiments. As shown in our results in Table 8 and Table 9 and Figure 9, snowy weather conditions exhibit relatively lower segmentation accuracy compared to other weather domains. Future work should incorporate unsupervised or semi-supervised training strategies to achieve higher performance in snowy conditions and further improve the quality of generated snowy scenes.

#### 5.3.2. Dataset and Resolution Limitations

Our experimental validation is subject to two primary data-related limitations. First, although we utilize the widely adopted ISPRS benchmark datasets [10,11], both Vaihingen and Potsdam represent similar German urban environments with comparable architectural styles and geographic characteristics. To enhance the robustness and generalization capability of our proposed system, we plan to extend our experiments to the LoveDA dataset [105], which comprises 5987 high-resolution aerial images from three distinct Chinese cities, encompassing both urban and rural scenarios. This expansion will provide more comprehensive validation across diverse geographic contexts and land-use patterns.

Second, due to computational constraints, our current experiments are conducted at 512×512 resolution, which is a standard protocol in semantic segmentation research. However, compared to higher-resolution imagery (e.g., 1024×1024), this resolution results in inevitable information loss. We have preliminarily tested diffusion models for 1024×1024 image-to-image translation, but the computational cost is prohibitively high—the saved checkpoint alone exceeds 10GB. Given current computational resource limitations, we are unable to conduct comprehensive studies at higher resolutions at this time. Nevertheless, generating higher-resolution weather domain data remains an important direction for future work.

### 5.4. Future Deployment and System Architecture

Looking forward, we aim to deploy our trained segmentation models on NVIDIA Jetson embedded devices mounted on drones for continuous city monitoring and surveillance applications, as well as for disaster response and humanitarian assistance missions. As demonstrated in our GitHub repository (https://github.com/WayBob/domainshifter, accessed on 31 October 2025), we are currently deploying our agent system as a Model Context Protocol (MCP) server [106]. This architecture enables remote invocation of the LLM agent from devices without CUDA-enabled GPUs, facilitating distributed deployment scenarios where edge devices can access centralized generative and segmentation capabilities through network connections. This design significantly reduces hardware requirements at deployment sites while maintaining the full functionality of our multi-weather domain transfer system.

## 6. Conclusions

In this paper, we have addressed the critical challenge of weather change-caused domain shift in aerial image segmentation by proposing **Multi-Weather DomainShifter**, a comprehensive multi-weather domain transfer system.

### 6.1. Technical Contributions and Experimental Validation

Our technical contributions encompass three core components:

First, we developed the **Aerial Weather Synthetic Dataset (AWSD)**, a controlled synthetic benchmark created using Unreal Engine that provides diverse weather conditions (overcast, foggy, dusty) for style reference. This dataset addresses the scarcity of multi-weather aerial imagery in existing benchmarks and serves as the foundation for domain-specific knowledge transfer.

Second, we proposed the **Latent Aerial Style Transfer (LAST)** model, which operates efficiently in the latent space to transfer atmospheric and illumination characteristics from synthetic references to real aerial images while preserving exact spatial layouts and semantic content. With computational efficiency of 9.45 FPS on a single RTX 4090, LAST enables large-scale data augmentation for atmospheric weather conditions.

Third, we introduced the **Multi-Modal Snowy Scene Diffusion Model (MSDM)**, which combines ControlNet-based structural conditioning with LLM-assisted scene descriptors to generate realistic snowy scenes with physical alterations. By leveraging Qwen3-14B for intelligent prompt generation from segmentation masks, MSDM ensures semantic consistency while adding complex weather-specific visual elements.

Fourth, we integrated these specialized generative models into a unified system coordinated by an LLM agent following the ReAct framework. This agent automatically decomposes complex domain transfer tasks, selects appropriate tools based on weather conditions, and manages the entire augmentation pipeline, demonstrating the system’s capacity for intelligent orchestration and practical scalability.

Extensive experiments on nine state-of-the-art segmentation models demonstrate the severe effects of weather change-caused domain shift, with average performance degradation of {3.35%, 3.92%, 10.55%, 28.59%} in mIoU under {overcast, foggy, dusty, snowy} conditions. Our ablation studies validate the effectiveness and cross-distribution generalization capability of synthetic weather data, while the comprehensive evaluation demonstrates substantial performance recovery: {+2.75%, +3.48%, +10.53%, +17.55%} in mIoU across shifted domains while maintaining competitive performance in the original domain.

### 6.2. Practical Significance and Real-World Implementation

Beyond technical novelty, this work addresses a fundamentally underestimated problem in the remote sensing and aerial image analysis community: **weather change-caused domain shift**. While existing research predominantly focuses on clear-weather scenarios, real-world deployment environments, including disaster response, environmental surveillance, and urban monitoring, frequently encounter adverse weather conditions that cause dramatic performance degradation in segmentation models.

Traditional approaches for handling weather-degraded imagery rely on separate preprocessing modules such as de-fogging, de-raining, and de-snowing algorithms. However, these auxiliary modules introduce significant practical challenges that limit their applicability in real-world geo-spatial tasks:

(1) Computational overhead and inference latency: Each additional preprocessing module increases the total computational FLOPs and extends the inference pipeline, which is particularly problematic for time-critical applications such as post-disaster assessment, real-time surveillance, and emergency response scenarios where rapid analysis is essential.

(2) Hardware burden: Deploying multiple weather-specific modules requires additional memory and processing resources, complicating system architecture and increasing deployment costs, especially for edge computing scenarios with resource-constrained devices (e.g., drones, robots, autonomous-driving cars, etc.)

(3) Sequential processing dependencies: Preprocessing modules create dependencies in the inference pipeline, where failures or artifacts in weather removal can propagate and degrade downstream segmentation performance.

In contrast, our **Multi-Weather DomainShifter** addresses these limitations through an end-to-end training paradigm. By generating diverse weather-augmented data with **AWSD**, **LAST**, and **MSDM**, we enable segmentation models to be inherently robust across all weather conditions without requiring any additional modules during inference. Critically, our generated data preserves exact spatial layouts and semantic content, **eliminating the need for additional manual annotation**. In particular, this is a significant advantage that drastically reduces the laborious and expensive annotation burden typical of domain adaptation approaches.

Furthermore, the entire data generation process is **automated and coordinated by an advanced LLM agent**, which intelligently selects appropriate generative tools (LAST for atmospheric changes, MSDM for physical alterations), manages batch processing, and ensures quality control. This automation enables scalable deployment across diverse geographical regions and weather scenarios without requiring extensive human intervention or domain expertise.

The practical convenience for real-world implementation is substantial: (1) Models trained with our augmented data can be deployed with identical inference costs as baseline models—no additional FLOPs, no extra latency, requiring only 6GB VRAM on consumer-grade GPUs; (2) The training process remains simple and direct supervised learning, leveraging standard optimization frameworks without complex adversarial training or domain adaptation algorithms; (3) The system architecture will be deployable as a Model Context Protocol (MCP) server, supporting distributed and scalable deployment from single-drone operations to city-wide monitoring networks.

As a result, this paper provides not merely a research contribution but a **deployable solution** that bridges the critical gap between laboratory benchmarks and practical applications in diverse environmental conditions, enabling robust and reliable aerial image analysis across the full spectrum of real-world weather scenarios.

## Figures and Tables

**Table 4:** Effect of Domain shift evaluated on Vaihingen dataset. mIoU (%) performance across different weather conditions.

Method		Weather Conditions				
**Model**	**Backbone**	**Original**	**Overcast**	**Foggy**	**Dusty**	**Snowy**
UperNet [15]	Swin-T [12]	73.26	68.27	66.66	66.46	43.54
UperNet	ResNet-50 [13]	73.33	68.47	68.18	56.16	42.52
UperNet	ViT-B	72.47	71.47	71.43	67.62	46.66
DeepLabv3+ [16]	ResNet-50	72.84	69.54	68.89	58.80	43.37
DANet [17]	ResNet-50	72.47	69.17	68.44	60.82	42.47
PointRend [18]	ResNet-50	72.67	69.56	69.48	57.71	42.58
FCN [19]	ResNet-50	72.79	67.78	66.81	61.98	43.62
Segmenter [20]	ViT-B	68.93	67.28	67.10	64.61	44.98
PSPNet [21]	ResNet-50	72.91	70.00	69.41	62.55	44.60
*Average*		72.41	69.06	68.49	61.86	43.82

**Table 5:** Effect of Domain shift evaluated on Vaihingen dataset. mFscore (%) performance across different weather conditions.

Method		Weather Conditions				
**Model**	**Backbone**	**Original**	**Overcast**	**Foggy**	**Dusty**	**Snowy**
UperNet [15]	Swin-T [12]	83.00	78.89	76.91	78.27	56.83
UperNet	ResNet-50 [13]	83.12	79.42	79.12	68.77	55.55
UperNet	ViT-B [14]	82.52	81.90	81.96	78.46	59.14
DeepLabv3+ [16]	ResNet-50	82.78	80.17	79.44	71.28	56.36
DANet [17]	ResNet-50	82.57	79.92	79.20	73.16	55.45
PointRend [18]	ResNet-50	82.77	80.46	80.36	70.20	55.56
FCN [19]	ResNet-50	82.84	78.63	77.41	74.15	56.39
Segmenter [20]	ViT-B	79.46	78.39	78.38	75.86	57.41
PSPNet [21]	ResNet-50	82.75	80.47	79.85	73.74	57.25
*Average*		82.42	79.81	79.18	73.77	56.66

**Table 6:** Ablation Study results for validating synthetic weather data effectiveness. All experiments use DeepLabv3+ with ResNet-50 backbone. mIoU (%) performance across different weather conditions.

Experiment		Weather Conditions				
**ID**	**Training Configuration**	**Original**	**Overcast**	**Foggy**	**Dusty**	**Snowy**
*Vaihingen Domain*						
Exp. 1	Vaihingen (VN) Ori	72.84	69.54	68.89	58.80	43.37
Exp. 2	+ VN Weather (w/o. snow)	73.69	72.97	73.29	73.11	46.18
Exp. 3	+ VN All Weather (w. snow)	73.35	72.20	72.36	72.90	62.76
*Potsdam Domain*						
Exp. 4	Potsdam Ori	74.07	68.77	69.09	40.75	40.27
Exp. 5	+ VN Original	74.34	65.82	65.10	50.94	40.16
Exp. 6	+ VN Weather (w/o. snow)	74.12	68.28	68.42	68.73	41.50
Exp. 7	+ VN All Weather (w. snow)	74.44	70.89	70.81	70.67	46.14

**Table 7:** Ablation study results for validating synthetic weather data effectiveness. All experiments use DeepLabv3+ with ResNet-50 backbone. mFscore (%) performance across different weather conditions.

Experiment		Weather Conditions				
**ID**	**Training Configuration**	**Original**	**Overcast**	**Foggy**	**Dusty**	**Snowy**
*Vaihingen Domain*						
Exp. 1	Vaihingen (VN) Ori	82.78	80.17	79.44	71.28	56.36
Exp. 2	+ VN Weather (w/o. snow)	83.85	83.21	83.47	83.29	58.41
Exp. 3	+ VN All Weather (w. snow)	83.81	82.96	83.08	83.39	73.80
*Potsdam Domain*						
Exp. 4	Potsdam Ori	83.72	79.69	79.89	54.03	54.46
Exp. 5	+ VN Original	84.03	77.18	76.73	64.23	54.30
Exp. 6	+ VN Weather (w/o. snow)	83.92	79.19	79.18	80.04	55.85
Exp. 7	+ VN All Weather (w. snow)	84.13	81.55	81.39	81.50	60.80

**Table 8:** Comprehensive results against Domain Shift on Vaihingen dataset. mIoU (%) performance across different weather conditions.

Method		Weather Conditions				
**Model**	**Backbone**	**Original**	**Overcast**	**Foggy**	**Dusty**	**Snowy**
UperNet [15]	Swin-T [12]	72.91	72.25	72.34	73.07	61.75
UperNet	ResNet-50 [13]	73.84	73.14	73.35	73.52	61.49
UperNet	ViT-B	72.80	72.03	72.24	73.10	63.20
DeepLabv3+ [16]	ResNet-50	73.35	72.20	72.36	72.90	62.76
DANet [17]	ResNet-50	72.44	72.06	72.44	72.81	61.34
PointRend [18]	ResNet-50	72.09	71.64	71.75	72.12	60.12
FCN [19]	ResNet-50	72.68	71.37	71.55	72.42	60.37
Segmenter [20]	ViT-B	69.38	68.86	68.94	68.96	59.68
PSPNet [21]	ResNet-50	73.07	72.76	73.01	73.13	61.64
*Average*		**72.51**	**71.81**	**72.00**	**72.45**	**61.37**

**Table 9:** Comprehensive results against Domain Shift on Vaihingen dataset. mFscore (%) performance across different weather conditions.

Method		Weather Conditions				
**Model**	**Backbone**	**Original**	**Overcast**	**Foggy**	**Dusty**	**Snowy**
UperNet [15]	Swin-T [12]	82.68	82.14	82.23	82.88	72.64
UperNet	ResNet-50 [13]	84.04	83.42	83.61	83.74	72.42
UperNet	ViT-B [14]	82.63	82.78	82.10	82.92	74.13
DeepLabv3+ [16]	ResNet-50	83.81	82.96	83.08	83.39	73.80
DANet [17]	ResNet-50	82.60	82.27	82.61	82.93	71.92
PointRend [18]	ResNet-50	82.68	82.32	82.40	82.69	71.65
FCN [19]	ResNet-50	82.94	81.91	82.07	82.69	71.34
Segmenter [20]	ViT-B	80.20	79.78	79.88	79.86	71.04
PSPNet [21]	ResNet-50	83.26	82.99	83.21	83.27	72.65
*Average*		**82.76**	**82.29**	**82.36**	**82.70**	**72.40**

## Data Availability

The original data presented in the study are openly available in the ISPRS Vaihingen and Potsdam datasets at https://www.isprs.org/ (accessed on 29 July 2025).

## Abbreviations

The following abbreviations are used in this manuscript:

AIS	Aerial Image Segmentation
AWSD	Aerial Weather Synthetic Dataset
LAST	Latent Aerial Style Transfer
MSDM	Multi-Modal Snowy Scene Diffusion Model
LLM	Large Language Model
VAE	Variational Autoencoder
GAN	Generative Adversarial Network
LDM	Latent Diffusion Model
DM	Diffusion Model
T2I	Text-to-Image
I2I	Image-to-Image
MSA	Multi-head Self-Attention
MCA	Multi-head Cross-Attention
FFN	Feed-Forward Network
FCN	Fully Convolutional Network
CNN	Convolutional Neural Network
ViT	Vision Transformer
ISPRS	International Society for Photogrammetry and Remote Sensing
mIoU	mean Intersection over Union
GSD	Ground Sampling Distance

## Appendix A

### Appendix A.1

The appendix provides more specific and spread experiment results for the ablation study in Section 4.5. In particular, it demonstrates detailed class segmentation performance across the weather change for Table 6 and Table 7. This extended results are in the following Table A1 and Table A2.

**Table A1:** Comprehensive per-class IoU (%) performance across all ablation experiments and weather conditions. DeepLabv3+ [16] with ResNet-50 backbone.

ID	Training Config	Weather	Imp. Surf.	Building	Low Veg.	Tree	Car	Clutter
*Vaihingen Domain*								
Exp. 1	VN Ori	Original	85.53	91.04	70.74	79.76	74.87	35.11
		Overcast	82.2	88.79	68.05	79.2	69.97	29.03
		Foggy	81.02	88.76	67.53	79.21	70.94	25.84
		Dusty	55.05	85.8	52.03	78.16	64.11	17.63
		Snowy	55.18	64.89	29.18	43.01	65.88	2.09
Exp. 2	+ VN Weather (w/o. snow)	Original	84.66	91.23	69.54	78.93	74.67	43.09
		Overcast	84.65	90.95	69.48	78.84	73.91	40.01
		Foggy	84.86	91.12	69.53	78.9	74.33	41.01
		Dusty	84.65	91.12	69.48	78.94	74.47	39.99
		Snowy	58.18	67.62	27.69	54.05	68.88	0.69
Exp. 3	+ VN All Weather (w. snow)	Original	82.76	90.13	67.44	79.35	74.01	46.42
		Overcast	82.61	89.64	67.61	78.86	70.23	44.26
		Foggy	82.57	89.31	67.76	78.95	71.18	44.42
		Dusty	83.14	89.86	68.62	79.38	72.64	43.74
		Snowy	74.67	84.41	55.45	70.99	74.31	16.75
*Potsdam Domain*								
Exp. 4	Potsdam Ori	Original	83.99	91.72	73.14	75.17	83.36	37.06
		Overcast	79.97	89.04	63.71	69.04	80.99	29.9
		Foggy	78.02	88.64	66.13	71.7	80.99	29.08
		Dusty	26.81	70.62	23.48	58.51	57.37	7.72
		Snowy	65.22	49.05	15.73	57.04	40.1	14.46
Exp. 5	+ VN Original	Original	83.77	91.67	73.18	75.48	82.91	39.03
		Overcast	74.36	89.04	56.01	69.25	80.81	25.44
		Foggy	71.61	88.16	52.72	70.62	81.0	26.47
		Dusty	44.3	76.9	40.87	59.63	72.79	11.14
		Snowy	65.58	49.94	15.27	55.95	39.89	14.34
Exp. 6	+ VN Weather (w/o. snow)	Original	83.76	91.67	72.61	74.98	82.3	39.42
		Overcast	78.05	89.01	64.45	70.77	79.78	27.6
		Foggy	77.4	89.5	65.26	71.77	80.12	26.45
		Dusty	76.46	85.59	65.68	71.44	80.01	33.2
		Snowy	66.61	52.04	18.11	55.58	41.7	14.95
Exp. 7	+ VN All Weather (w. snow)	Original	83.94	91.69	72.7	75.15	83.34	39.8
		Overcast	80.97	89.6	66.85	70.67	81.81	35.46
		Foggy	79.84	89.93	66.73	72.22	82.11	34.03
		Dusty	77.67	88.16	67.32	72.81	82.07	36.02
		Snowy	69.83	56.58	30.66	63.44	38.37	17.96

**Table A2:** Comprehensive per-class F-score (%) performance across all ablation experiments and weather conditions. DeepLabv3+ [16] with ResNet-50 backbone.

ID	Training Config	Weather	Imp. Surf.	Building	Low Veg.	Tree	Car	Clutter
*Vaihingen Domain*								
Exp. 1	VN Ori	Original	92.2	95.31	82.86	88.74	85.63	51.97
		Overcast	90.23	94.06	80.99	88.39	82.34	45.00
		Foggy	89.52	94.05	80.62	88.4	83.0	41.07
		Dusty	71.01	92.36	68.45	87.74	78.13	29.98
		Snowy	71.09	78.66	44.95	59.96	79.43	4.09
Exp. 2	+ VN Weather (w/o. snow)	Original	91.7	95.41	82.04	88.23	85.5	60.23
		Overcast	91.69	95.26	81.99	88.17	85.0	57.15
		Foggy	91.81	95.35	82.03	88.21	85.28	58.17
		Dusty	91.69	95.35	81.99	88.23	85.36	57.13
		Snowy	73.55	80.67	43.16	70.13	81.57	1.37
Exp. 3	+ VN All Weather (w. snow)	Original	90.57	94.81	80.55	88.48	85.06	63.4
		Overcast	90.48	94.54	80.68	88.18	82.51	61.36
		Foggy	90.45	94.35	80.78	88.23	83.16	61.52
		Dusty	90.79	94.66	81.39	88.5	84.15	60.86
		Snowy	85.43	91.52	71.11	82.96	85.25	26.56
*Potsdam Domain*								
Exp. 4	Potsdam Ori	Original	91.3	95.68	84.49	85.82	90.92	54.08
		Overcast	88.87	94.2	77.83	81.69	89.5	46.03
		Foggy	87.65	93.98	79.61	83.52	89.5	45.05
		Dusty	42.28	82.78	38.03	73.83	72.91	14.33
		Snowy	78.93	65.79	27.01	72.61	57.22	25.18
Exp. 5	+ VN Original	Original	91.17	95.66	84.52	86.03	90.66	56.15
		Overcast	85.3	94.2	71.8	81.83	89.39	40.56
		Foggy	83.45	93.71	69.04	82.78	89.5	41.86
		Dusty	61.4	86.94	58.02	74.71	84.25	20.04
		Snowy	79.2	66.58	26.34	71.72	57.01	24.97
Exp. 6	+ VN Weather (w/o. snow)	Original	91.16	95.66	84.13	85.7	90.29	56.55
		Overcast	87.67	94.18	78.38	82.89	88.75	43.27
		Foggy	87.26	94.46	78.98	83.56	88.96	41.84
		Dusty	86.66	92.24	79.29	83.34	88.9	49.85
		Snowy	79.94	68.43	30.5	71.41	58.83	25.96
Exp. 7	+ VN All Weather (w. snow)	Original	91.27	95.67	84.19	85.81	90.91	56.94
		Overcast	89.48	94.52	80.13	82.82	90.0	52.35
		Foggy	88.79	94.7	80.04	83.87	90.17	50.78
		Dusty	87.43	93.71	80.47	84.27	90.15	52.96
		Snowy	82.21	72.24	46.88	77.62	55.42	30.41

#### Appendix A.1.1. Per-Class Performance Analysis: Intra-Distribution Validation

The per-class results in Table A1 and Table A2 reveal heterogeneous vulnerability patterns across semantic categories under weather change-caused domain shifts. In the baseline Vaihingen [10,11] experiment (Exp. 1), *Impervious Surfaces* (including roads, parking lots) suffers the most severe degradation under dusty conditions, dropping from **85.53%** to **55.05%** IoU (**−30.48%**), as atmospheric obscuration severely affects texture and color dependent surface recognition. In contrast, *Buildings* maintains relative stability at **85.80%** IoU (**−5.24%**), attributable to their distinct appearance features and structural geometry that remain recognizable despite atmospheric changes. Snowy conditions induce complete color transformations of target objects, causing dramatic domain shift effects: *Clutter* (background elements) drops from **35.11%** to **2.09%** IoU (**−33.02%**), *Low Vegetation* drops from **70.74%** to **29.18%** (**−41.56%**), and *Tree* decreases from **79.76%** to **43.01%** (**−36.75%**), as snow coverage fundamentally alters their visual appearance from green/brown to white. With atmospheric weather augmentation (Exp. 2), *Low Vegetation* improves dramatically under dusty conditions from **52.03%** to **69.48%** IoU (**+17.45%**), and *Clutter* shows consistent gains (dusty: **+22.36%**). When incorporating snow data (Exp. 3), natural elements recover substantially: *Low Vegetation* improves from **29.18%** to **55.45%** IoU (**+26.27%**), and *Tree* from **43.01%** to **70.99%** IoU (**+27.98%**), demonstrating that our MSDM model successfully learns snow related appearance transformations.

##### Per-Class Performance Analysis: Cross-Distribution Validation

The Potsdam [10,11] domain experiments (Exp. 4–7) provide class-level evidence of cross-distribution knowledge transfer. In Exp. 4 baseline, *Impervious Surfaces* under dusty conditions shows extreme vulnerability at **26.81%** IoU, significantly worse than Vaihingen (Exp. 1: **55.05%**), indicating geographical variations in road surface textures between urban regions. The critical Exp. 5 reveals that adding only original Vaihingen data without weather diversity causes class-specific degradation: *Impervious Surfaces* under overcast drops from **79.97%** to **74.36%** (**−5.61%**), and *Low Vegetation* from **63.71%** to **56.01%** (**−7.70%**), as models trained on clear-weather data from multiple regions fail to learn weather-specific features. With synthetic weather data (Exp. 6), texture-dependent classes recover dramatically: *Impervious Surfaces* under dusty conditions improves from **26.81%** to **76.46%** IoU (**+49.65%**), and *Low Vegetation* gains from **23.48%** to **65.68%** (**+42.20%**), demonstrating that weather-specific training data enables models to recognize surfaces despite atmospheric obscuration. The comprehensive augmentation (Exp. 7) further enhances color-sensitive classes under snowy conditions: *Low Vegetation* improves from **15.73%** to **30.66%** (**+14.93%**), and *Buildings* from **49.05%** to **56.58%** (**+7.53%**), where the latter’s smaller improvement reflects its reliance on structural features that remain relatively invariant to color changes. These results confirm that structural classes benefit from geometric consistency across weather conditions, while natural elements require explicit training on appearance transformations caused by snow coverage.
